# Impact of combined training with different exercise intensities on inflammatory and lipid markers in type 2 diabetes: a secondary analysis from a 1-year randomized controlled trial

**DOI:** 10.1186/s12933-020-01136-y

**Published:** 2020-10-07

**Authors:** João P. Magalhães, Diana A. Santos, Inês R. Correia, Megan Hetherington-Rauth, Rogério Ribeiro, João F. Raposo, Andreia Matos, Manuel D. Bicho, Luís B. Sardinha

**Affiliations:** 1 Exercise and Health Laboratory, CIPER, Faculdade de Motricidade Humana, Universidade de Lisboa, Estrada da Costa, 1499-002 Cruz-Quebrada, Portugal; 2 Education and Research Centre, APDP–Diabetes Portugal (APDP-ERC), Rua Rodrigo da Fonseca 1, 1250-189 Lisbon, Portugal; 3 Genetics Laboratory Environmental Health Institute (ISAMB), Faculty of Medicine, University of Lisbon, Avenida Professor Egas Moniz MB, 1649-028 Lisbon, Portugal; 4 Instituto de Investigação Científica Bento da Rocha Cabral, Calçada Bento da Rocha Cabral 14, 1250-012 Lisbon, Portugal; 5 Tumor & Microenvironment Interactions Group i3S -Instituto de Investigação e Inovação em Saúde/INEB-Institute of Biomedical Engineering, University of Porto, Rua Alfredo Allen, 208, 4200-135 Porto, Portugal; 6 Abel Salazar Institute for the Biomedical Sciences (ICBAS), University of Porto, Rua de Jorge Viterbo Ferreira No. 228, 4050-313 Porto, Portugal

**Keywords:** High-intensity interval training, Moderate continuous training, Exercise, Resistance training

## Abstract

**Background:**

Exercise is a well-accepted strategy to improve lipid and inflammatory profile in individuals with type 2 diabetes (T2DM). However, the exercise intensity having the most benefits on lipids and inflammatory markers in patients with T2DM remains unclear. We aimed to analyse the impact of a 1-year combined high-intensity interval training (HIIT) with resistance training (RT), and a moderate continuous training (MCT) with RT on inflammatory and lipid profile in individuals with T2DM.

**Methods:**

Individuals with T2DM (n = 80, aged 59 years) performed a 1-year randomized controlled trial and were randomized into three groups (control, n = 27; HIIT with RT, n = 25; MCT with RT, n = 28). Exercise sessions were supervised with a frequency of 3 days per week. Inflammatory and lipid profiles were measured at baseline and at 1-year follow-up. Changes in inflammatory and lipid markers were assessed using generalized estimating equations.

**Results:**

After adjusting for sex, age and baseline moderate-to-vigorous physical activity (MVPA), we observed a time-by-group interaction for Interleukin-6 (IL-6) in both the MCT with RT (β = − 0.70, p = 0.034) and HIIT with RT (β = − 0.62, p = 0.049) groups, whereas, only the HIIT with RT group improved total cholesterol (β = − 0.03, p = 0.045) and LDL-C (β = − 0.03, p = 0.034), when compared to control. No effect was observed for C-reactive protein (CRP), cortisol, tumour necrosis factor-α (TNF-α), soluble form of the haptoglobin-hemoglobin receptor CD163 (sCD163), triglycerides and HDL-C in both groups (p > 0.05).

**Conclusions:**

Favorable adaptations on IL-6 were observed in both the HIIT and MCT combined with RT groups following a long-term 1-year exercise intervention in individuals with T2DM. However, only the HIIT with RT prevented further derangement of total cholesterol and LDL-C, when compared to the control group. Therefore, in order to encourage exercise participation and improve inflammatory profile, either exercise protocols may be prescribed, however, HIIT with RT may have further benefits on the lipid profile.

*Trial registration* Clinicaltrials.gov ID: NCT03144505

## Background

Individuals with type 2 diabetes (T2DM) have a heightened risk of all-cause mortality and cardiovascular disease (CVD), especially those with traditional CVD risk factors, such as hypertension, hyperglycaemia, abdominal obesity, and dyslipidaemia [1]. Low-grade systemic inflammation has been suggested as the common denominator linking T2DM, insulin resistance, endothelial dysfunction, metabolic syndrome, and CVD [2]. When released by adipose tissue as adipokines, tumour necrosis factor-α (TNF-α) and interleukin-6 (IL-6) promote low-grade systemic inflammation, which in turn is associated with chronic deleterious conditions such as insulin resistance, T2DM and obesity [2–5]. Another biomarker, connecting low-grade inflammation and T2DM, is the soluble form of the haptoglobin-hemoglobin receptor CD163 (sCD163), with observational studies suggesting that individuals with obesity have increased concentrations of sCD163 [6] putting them at higher risk for T2DM [7].

Exercise, especially the combination of both aerobic and resistance training (RT) [8], has many health benefits for patients with T2DM, including improved body composition [9, 10], insulin sensitivity [10–12], lipid profile, and reduced low-grade systemic inflammation [13]. However, exercise permutations, such as in the duration and intensity of the exercise performed, may influence the inflammatory and lipid profile differently. Recently, high-intensity interval training (HIIT) has emerged as a viable alternative in several conditions including those with CVD [14], obesity [15], and diabetes [16], while having the same or even superior health benefits, to that of the more commonly performed exercise modality of moderate continuous training (MCT) [15–17]. In fact, a previous review with T2DM individuals suggests that short to medium-term HIIT interventions improve glycaemic control, lipid profile, body composition, and cardiorespiratory fitness [18]. However, the same review suggested that there was no consensus on the long-term effects of HIIT, nor the effectiveness of HIIT vs. MCT on lipid profile in individuals with T2DM, mainly owing to the inexistence of longer interventions or the reduced number of randomized control trials (RCT) analysing both protocols. As far as the inflammatory profile goes, just two non-RCTs analysed the impact of HIIT in individuals with T2DM, with both having a short intervention duration (< 12-weeks) and with the results suggesting limited impact [19, 20]. Given that currently the information related with HIIT and its impact on both the lipid and inflammatory profile is derived from short to medium-term investigations (≤ 24-weeks), it is paramount to understand if these previous findings can be replicated in longer-term controlled exercise interventions, as HIIT has been shown to be a highly demanding type of exercise [21].

To the best of our knowledge, no investigation has analysed the long-term impact of combined aerobic exercise of different intensities with RT on both the inflammatory and lipid profile in individuals with T2DM. Therefore, the present investigation aimed to analyse the effects of a 1-year RCT consisting of a control, a combined MCT with RT, and a combined HIIT with RT group, on the inflammatory and lipid profile in individuals with T2DM.

## Methods

### Participants, randomization, and blinding

This investigation was part of a larger RCT performed between February 2014 to July 2016 at the Exercise and Health Laboratory, Faculty of Human Kinetics, University of Lisbon, and was carried out following the recommendations of the Declaration of Helsinki for Human Studies. The protocol was approved by the Ethics Committee of the Portuguese Diabetes Association (approval number: 07/17/2013). Written informed consents were obtained from all participants.

**Fig. 1:**
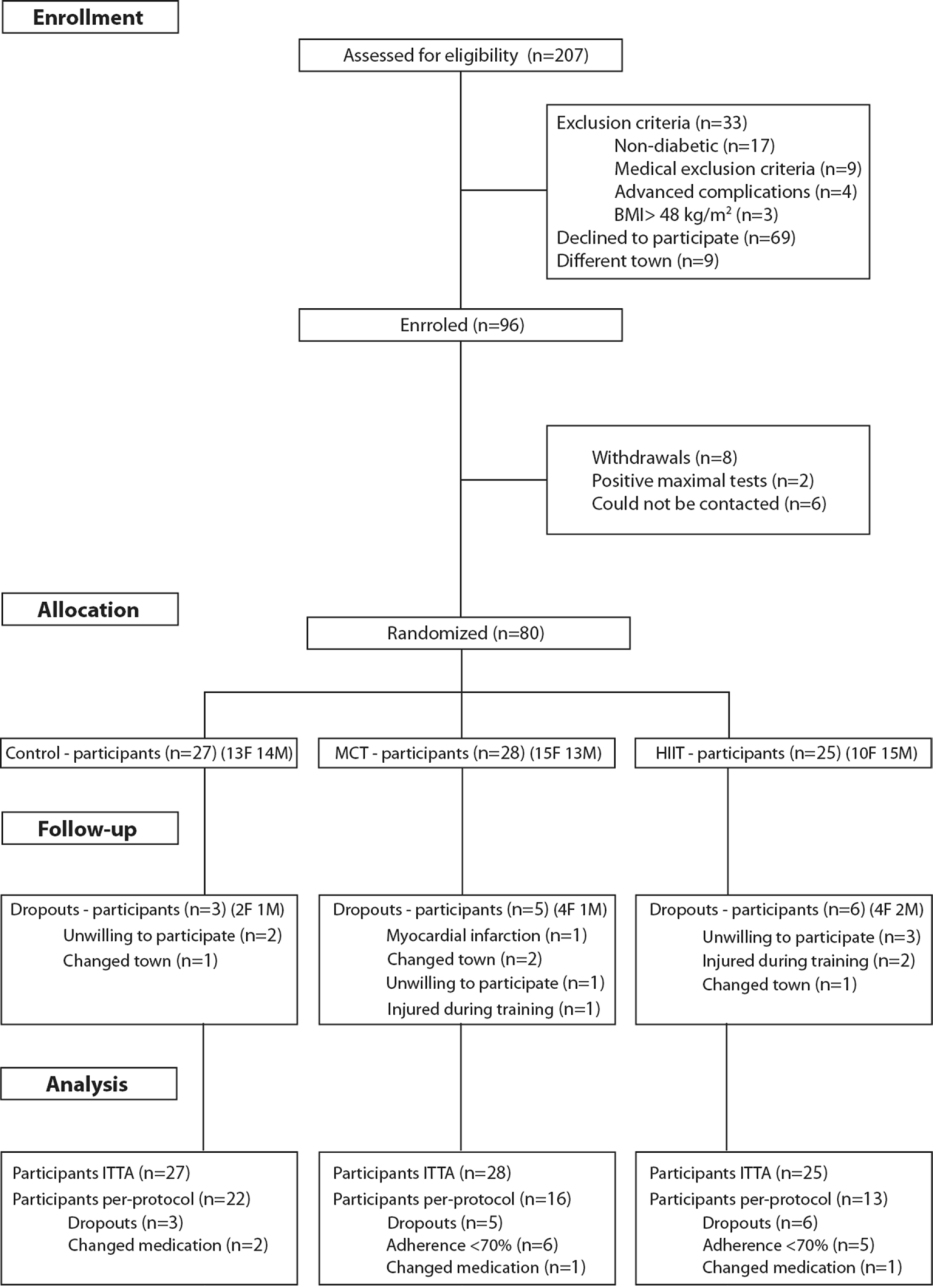
Study flow chart

The complete study protocol has been previously published [22]. Briefly, individuals with T2DM were recruited to analyse the impact of a 1-year exercise intervention with different intensities on glycated haemoglobin (HbA1c) (clinicaltrials.gov ID:NCT03144505). Eighty patients were recruited within the Lisbon Metropolitan Area (Fig. 1) [22], and posteriorly randomized into three groups (control, HIIT with RT or MCT with RT). An external researcher, with an allocation ratio of 1:1:1, used a computer-generated list of random numbers, for the randomization process. The researchers performing the assessments were blinded to group randomization. Inclusion criteria for participants included adults diagnosed with T2DM [23], age between 30 and 75 years old, no major micro or macro vascular complications from diabetes, body mass index < 48 kg/m^2^, and no limitations that would prevent them from practicing exercise. The main outcome power and sample size calculations (G-Power, Version 3.1.3) were based on a predicted HbA1c difference of 0.66% with a SD of 1.2%, α = 0.05, 1-β = 0.80 and an expected dropout rate of 10% [24]. For this analysis, the power and sample size calculations were based on changes in whole-body fat, which is related with overall inflammatory profile. Given a predicted whole-body fat difference of 2.7%, with a SD of 1.7%, α = 0.05, 1-β = 0.80, the sample used on this study was powered for this analysis [16].

The control group had an initial standard physical activity (PA) recommendation session and no structured exercise. All of the exercise groups (i.e. the MCT and HIIT group) had three supervised exercise sessions per week, monitored with a heart rate polar band (Polar T-31, USA). The exercise programs of both groups were developed to have matched energy expenditure, with a weekly target of 10 kcal/kg, which was updated monthly for their body weight and every 3 months for their peak oxygen consumption.

The MCT and HIIT groups had an exercise periodization for the 1-year divided in two and three phases, respectively. Heart rate reserve (HRR), calculated through the Karvonen formula [25], was used to achieve prescribed intensities. Phase 1 was identical for both groups (weeks 1–4), with patients performing continuous cycling of moderate-intensity (40–60% of the HRR) with durations increasing from 15 min to 25 by the end of week 4. The MCT group had only one additional phase (training phase, weeks 5–52), where participants exercised at 40 to 60% of the HRR, with durations based on prescribed energy expenditure targets.

In the HIIT group, during phase 2 (5–8 weeks), patients performed bouts of 2 min of cycling at 70% of the HRR followed by 1 min at 40–60% of the HRR (weeks 5–6), and increased to bouts of 80% (1.5 min) of the HRR followed by 1 min at 40–60% of the HRR (weeks 7–8), while maintaining energy expenditure targets. In phase 3 (weeks 9–52), participants in the HIIT group performed 1 min of exercise at 90% of their HRR followed by 1 min resting at 40-60% of the HRR. Both the MCT and HIIT group were further complemented with a whole-body RT, after the aerobic component, which included 1 set of 10–12 RM of eight exercises (seated row, pulldown, chest press, shoulder press, leg press, one leg lung, dead bug and regular plank).

### Anthropometry and body composition

Patients were weighed on an electronic scale, to the nearest 0.01 kg while wearing minimal clothes (Seca, Hamburg, Germany). Height was measured to the nearest 0.1 cm with a stadiometer (Seca, Hamburg, Germany). Waist circumference was taken according to the standardized procedures of the National Institute of Health [26].

Dual energy X-ray absorptiometry (Hologic Explorer-W, Waltham, USA) was used to assess regional and total body fat, following standardized protocols and procedures set out by the manufacturer. Whole-body fat index (WBFI) and abdominal fat index (AFI) were calculated by dividing the total and abdominal fat mass by the square of the height (kg/m^2^).

### Objective measures of moderate-to-vigorous physical activity

Moderate-to-vigorous PA (MVPA) was assessed by accelerometry (ActiGraph, GT3X + , FL, USA) at baseline prior to the start of the intervention. All participants used the accelerometer for 7 days, on the right hip. The devices were activated on raw mode with a 100 Hz frequency and later transformed into 15-s epochs. The Troiano et al. [27] cut points and wear time validation criteria were used.

### Laboratory measurements

Blood collection was performed in a seated position from the antecubital vein at rest after an overnight fast into dry tubes and into tubes containing ethylenediamine-tetraacetic acid as an anticoagulant. Biological samples were centrifuged at 500*g* at 4 °C for 15-min and plasma samples were frozen at − 80 °C for posterior analysis.

Serum samples were used to analyse the lipid profile of the participants, including the quantification of total cholesterol, LDL-C and HDL-C cholesterol, and triglycerides using colored enzymatic tests in an automated analyser (auto analyser Olympus AU640, Beckman Coulter). Plasma samples were then used for TNF- α, IL-6, sCD163, C-reactive protein (CRP), and cortisol quantification using commercial ELISA kits (DiaSource Immuno Assays S.A for TNF-α, IL6, and Cortisol; IBL International GMBH for CRP; and DC1630, R&D Systems for sCD163).

Changes in the lipid and inflammatory profile were analyzed at baseline and at the 1-year follow-up.

### Statistical analysis

Data analyses were performed using SPSS Statistics version 22.0 (SPSS Inc., an IBM Company, Chicago, Illinois, USA). Results are presented as mean ± SD for all normally distributed outcomes and as median and inter-quartile range for skewed outcomes. Comparisons between groups were performed using the Chi-squared test for sex proportions between groups, and the parametric independent sample ANOVA test with a Bonferroni post hoc analysis for normally distributed variables or the non-parametric Kruskal–Wallis test in absence of normality.

Between-group and within-group effects for the lipid inflammatory profile were performed using generalized estimating equations followed by a least significant difference post hoc test. Models were adjusted for potential confounders (i.e. age, sex and baseline MVPA). All the outcomes went through an intention-to-treat analysis (ITTA). An additional per-protocol analysis (PPA) was performed in only those who completed both assessments (i.e. baseline and 1-year), had at least 70% attendance to all the exercise sessions, and in those without substantial changes in pharmacological therapy [22]. No changes were made to dyslipidemia and hypertension medication, however, individuals with major changes in anti-hyperglycemic medication, such as transitioning to insulin, were removed from the (PPA).

## Results

Table 1 shows the baseline characteristics of the individuals by intervention group and in the ITTA and PPA. No significant differences between groups were observed at baseline in both analyses, except for baseline time spent in MVPA (Table 1). For the control, MCT and HIIT groups, the dropout rates were 11%, 18%, and 24%, respectively (Fig. 1). In the PPA, individuals in the MCT and HIIT group trained for 45.0 ± 7.1 min/session and 33.1 ± 6.4 min/session, and had mean percent training adherences of 86.2% and 86.8%, respectively. Three individuals reported injuries during the intervention, two in the HIIT group while performing the leg-press exercise and one from the MCT group while performing the back-row exercise. The cardiovascular event reported in the flow chart took place during the participant’s day-to-day routine and was not related to the intervention.

**Table 1:** Baseline characteristics of the participants by group and all sample

	Intention to treat baseline values				Per-protocol baseline values			
	Control (n = 27)	MCT (n = 28)	HIIT (n = 25)	p-value	Control (n = 22)	MCT (n = 16)	HIIT (n = 13)	p-value
Age (years)	59.0 ± 8.1	59.7 ± 6.5	56.7 ± 8.3	0.575	60.8 ± 7.5	60.4 ± 6.8	58.9 ± 7.5	0.814
Woman, no (%)	48.1	53.6	40.0	0.612	50.0	56.3	30.8	0.367
Diabetes Diagnosis (years)^†^	5.0 ± 3.0	8.0 ± 9.0	5.0 ± 6.0	0.086	4.5 ± 3.25	8.0 ± 9.0	6.0 ± 6.0	0.091
Hypertension medication (%)	48.1	50.0	52.0	0.579	54.5	37.5	53.8	0.538
Oral antidiabetic medication (%)	96.3	92.9	84.0	0.388	95.5	93.8	92.3	0.512
Lipid lower medication (%)	33.3	25.0	24.0	0.596	31.8	31.3	23.1	0.341
Weight (kg)	84.1 ± 15.8	82.7 ± 13.3	81.6 ± 16.8	0.906	85.9 ± 15.7	82.0 ± 13.8	84.2 ± 19.2	0.799
Height (cm)	165.5 ± 9.4	163.2 ± 8.4	164.8 ± 8.1	0.545	164.2 ± 9.5	162.9 ± 9.2	166.6 ± 8.1	0.615
BMI (kg/m^2^)	30.7 ± 5.0	31.1 ± 5.0	30.1 ± 5.7	0.722	31.7 ± 4.7	31.0 ± 5.5	30.2 ± 5.9	0.506
WC (cm)	103.0 ± 12.4	103.8 ± 11.3	102.7 ± 14.3	0.914	105.4 ± 11.2	103.5 ± 12.2	103.3 ± 15.8	0.946
WB Fat Index (kg/m^2^)	10.5 ± 3.3	11.0 ± 3.4	10.0 ± 3.9	0.725	11.2 ± 2.9	11.0 ± 3.4	10.1 ± 3.9	0.662
AFI (kg/m^2^)	1.0 ± 0.4	1.1 ± 0.4	1.0 ± 0.4	0.680	1.1 ± 0.3	1.1 ± 0.4	1.0 ± 0.4	0.843
MVPA (min/day)^†^	18.4 ± 26.4	30.5 ± 4.8	38.9 ± 29.4	0.008*	15.9 ± 23.1	38.1 ± 41.6	38.9 ± 30.1	0.012*
HbA1c (mmol/mol)^†^	49.7 ± 20.7	53.2 ± 22.7	49.0 ± 12.3	0.545	48.1 ± 16.7	47.2 ± 22.1	50.8 ± 12.6	0.828
HbA1c (%)^†^	7.4 ± 1.8	7.4 ± 1.9	7.0 ± 1.1	0.545	6.9 ± 1.1	6.9 ± 1.6	6.9 ± 0.9	0.828
VO_2peak_ (ml/kg/min)	25.9 ± 5.5	24.1 ± 3.2	27.1 ± 6.3	0.143	25.1 ± 5.6	23.9 ± 3.7	26.6 ± 5.3	0.345

*AFI* android fat index, *BMI* body mass index, *HbA1c* glycated hemoglobin, *HIIT* high-intensity interval training, *MCT* moderate continuous training, *MVPA* moderate-to-vigorous physical activity, *WC* waist circumference, *WBFI* whole-body fat index

^*^Differences between group baseline values (p < 0.05)

†Skewed values are presented as median ± inter quartile range

Table 2 presents the body composition, the inflammatory (IL-6, TNF-α, CRP, sCD163 and cortisol) and lipid (LDL-C, HDL-C, and triglycerides) profile outcomes assessed at baseline and at follow-up by group, as well as the respective time-by-group interactions between each intervention group (MCT vs. HIIT vs. control) using the ITTA. All models were adjusted for sex, age, and baseline MVPA. Following adjustments, the ITTA analysis suggested that the HIIT prevented further derangement on total cholesterol (β = − 0.03, p = 0.045) and LDL-C (β = − 0.03, p = 0.034) when compared to the control, whereas the MCT had no effect on the lipid profile variables (p > 0.05). Only the MCT group had changes in WBFI (β = − 0.06, p = 0.025) and AFI (β = − 0.01, p = 0.011). Regarding the inflammatory profile, both the MCT (β = − 0.70, p = 0.034) and HIIT (β = − 0.62, p = 0.049) prevented further derangement (p < 0.05) in the levels of IL-6 following the 1-year intervention, when compared to controls. For the remaining inflammatory variables, no changes (> 0.05) were observed for both intervention groups compared to controls including the sCD163 biomarker. Moreover, there was no time-by-group interaction (p > 0.05) in any of the biomarkers measured when comparing both exercise groups (i.e. HIIT vs MCT).

**Table 2:** Intention-to-treat analysis for the lipid and inflammatory profile at baseline and following 1-year

	Control (n = 27)			MCT (n = 28)			HIIT (n = 25)			MCT* control	HIIT* control	MCT* HIIT
Outcome	Baseline	12 months	Δ	Baseline	12 months	Δ	Baseline	12 months	Δ	β (95% CI)	β (95% CI)	β (95% CI)
TC (mmol/L)	4.8 ± 1.0	5.2 ± 1.1^†^	0.4 ± 0.8	4.9 ± 1.4	4.9 ± 1.3	0.0 ± 0.7	5.0 ± 1.0	4.9 ± 0.8	− 0.1 ± 0.6	− 0.02 (− 0.06; 0.01)	− 0.03 (− 0.06; − 0.00)*	0.04 (− 0.02; 0.03)
LDL-C (mmol/L)	3.4 ± 0.8	3.6 ± 1.0	0.2 ± 0.7	3.5 ± 1.2	3.5 ± 1.1	− 0.0 ± 0.6	3.6 ± 0.8	3.5 ± 0.7	− 0.1 ± 0.4	− 0.03 (− 0.05; 0.00)	− 0.03 (− 0.05; − 0.00)*	0.00 (− 0.02; 0.02)
HDL-C (mmol/L)	1.2 ± 0.2	1.2 ± 0.3	0.0 ± 0.1	1.2 ± 0.3	1.3 ± 0.3	0.1 ± 0.2	1.2 ± 0.3	1.3 ± 0.2^†^	+0.1 ± 0.1	0.00 (− 0.00; 0.01)	0.00 (− 0.01; 0.01)	− 0.00 (− 0.01; 0.00)
Triglycerides (mmol/L)^+^	1.5 ± 1.0	1.6 ± 1.2	0.1 ± 1.1	1.4 ± 1.2	1.3 ± 1.2	− 0.1 ± 0.7	1.3 ± 1.2	1.5 ± 0.9	0.2 ± 0.7	− 0.02 (− 0.05; 0.02)	− 0.02 (− 0.05; 0.02)	− 0.00 (− 0.03; 0.03)
sCD163 (ng/L)	1.03 ± 0.4	1.00 ± 0.37	− 0.03 ± 0.3	0.89 ± 0.4	1.10 ± 0.4	0.21 ± 0.4	0.99 ± 0.4	0.98 ± 0.4	− 0.01 ± 0.3	− 0.01 (− 0.02;0.01)	0.00 (− 0.01;0.02)	− 0.01 (− 0.02; 0.01)
TNF-α (pg/ml)^+^	0.10 ± 0.17	0.99 ± 1.16^†^	− 0.01 ± 0.7	0.09 ± 0.10	0.06 ± 1.12	− 0.3 ± 0.8	0.10 ± 0.29	0.05 ± 1.07	− 0.05 ± 0.7	− 0.02 (− 0.05; 0.01)	− 0.03 (− 0.06; 0.00)	0.01 (− 0.02; 0.03)
IL-6 (pg/ml)	19.6 ± 13.4	25.7 ± 14.4^†^	6.0 ± 17.0	25.2 ± 14.3	23.1 ± 11.8	− 2.4 ± 13.6	19.9 ± 14.8	18.6 ± 10.8	− 1.3 ± 12.5	− 0.70 (− 1.35; − 0.05)*	− 0.62 (− 1.24; − 0.00)*	− 0.08 (− 0.62; 0.46)
CRP (nmol/L)^+^	32.3 ± 61.0	29.7 ± 35.3	− 2.6 ± 32.4	21.0 ± 34.7	23.9 ± 55.1	2.9 ± 35.0	29.7 ± 36.9	21.9 ± 59.3	− 7.8 ± 54.7	0.86 (− 0.57; 2.28)	0.92 (− 0.90; 2.73)	− 0.06 (− 1.91; 1.78)
Cortisol (nmol/L)^+^	398.0 ± 174.4	391.4 ± 264.3	− 6.6 ± 307.7	466.5 ± 358.2	368.9 ± 200.3	− 97.6 ± 414.5	408.4 ± 357.0	402.2 ± 251.4	− 6.6 ± 307.7	− 1.31 (− 16.7; 14.1)	− 1.90 (− 14.9; 11.1)	0.59 (− 14.7; 15.9)
WBFI (kg/m^2^)	10.5 ± 3.3	10.7 ± 3.5	0.2 ± 0.9	11.0 ± 3.4	10.5 ± 3.5†	− 0.5 ± 1.3	10.0 ± 3.9	9.7 ± 4.1	− 0.3 ± 0.8	− 0.06 (− 0.12; − 0.01)*	− 0.04 (− 0.09; 0.01)	− 0.02 (− 0.07; 0.03)
AFI (kg/m^2^)	1.0 ± 0.4	1.0 ± 0.4	0.0 ± 0.2	1.1 ± 0.4	1.0 ± 0.4†	− 0.1 ± 0.2	1.0 ± 0.4	1.0 ± 0.4	− 0.0 ± 0.1	− 0.01 (− 0.02; − 0.01)*	− 0.01 (− 0.01; 0.02)	− 0.01 (− 0.01; 0.00)
WC (cm)	103.0 ± 12.4	102.7 ± 11.8	− 0.3 ± 4.4	103.8 ± 11.3	101.8 ± 12.1	− 2.0 ± 4.9	102.7 ± 14.3	101.6 ± 14.4	− 1.1 ± 5.0	− 0.17 (− 0.44; 0.09)	− 0.08 (− 0.31; 0.15)	− 0.08 (− 0.32; 0.16)
Weight (kg)	84.1 ± 15.8	84.7 ± 15.2	0.6 ± 4.1	82.7 ± 13.4	82.0 ± 14.5	− 0.7 ± 5.1	81.6 ± 16.8	81.0 ± 17.0	− 0.6 ± 3.6	− 0.12 (− 0.34; 0.10)	− 0.08 (− 0.27; 0.12)	− 0.04 (− 0.26; 0.18)

Betas coefficients are presented as unstandardized coefficients adjusted for age, sex and baseline MVPA with the respective 95% confidence intervals

*AFI* android fat index, *CRP*, C-reactive protein, *HIIT* high-intensity interval training, *HDL-C* high-density lipoprotein cholesterol, *IL-6* interleukin 6; LDL-C low-density lipoprotein cholesterol, *MCT* moderate continuous training, *TC* total cholesterol, *TNF-α* tumor necrosis factor alfa, *WBFI* whole-body fat index

^*^ Between-group changes significant at p < 0.05

† Within-group changes significant at p < 0.05

^+^Skewed values are presented as median ± inter quartile range

Table 3 summarizes the results of the PPA for the lipid, inflammatory and body composition profile. As a result of the 1-year HIIT and MCT intervention, the HIIT group prevented further deterioration of the LDL-C profile (β = − 0.03, p = 0.049), when compared to control. On the other hand, no changes were observed between both groups and control for the remaining lipid profile variables, including total cholesterol, HDL-C, and triglycerides (p > 0.05). Within the inflammatory profile, IL-6 followed the same trend as in the ITTA for the MCT group (β = − 0.89, p = 0.047), while there were no changes for the HIIT group (β = − 0.72, p = 0.081). For both intervention groups, there was no time-by-group interaction (p > 0.05) for cortisol, TNF-α, sCD163, and CRP, when compared to control. Lastly, in line with the results from the ITTA, no time-by-group interaction (p > 0.05) was found between MCT and HIIT for any of the biomarkers assessed.

**Table 3:** Lipid and inflammatory profile at baseline and following 1-year using the per-protocol analysis

	Control (n = 27)			MCT (n = 28)			HIIT (n = 25)			MCT* Control	HIIT* Control	MCT* HIIT
Outcome	Baseline	12 months	Δ	Baseline	12 months	Δ	Baseline	12 months	Δ	β (95% CI)	β (95% CI)	β (95% CI)
TC (mmol/L)	4.8 ± 0.9	5.2 ± 1.1†	0.4 ± 0.9	4.5 ± 0.9	4.6 ± 0.9	0.1 ± 0.7	5.0 ± 1.1	4.9 ± 0.9	− 0.1 ± 0.6	− 0.00 (− 0.05; 0.04)	− 0.02 (− 0.07; 0.03)	0.01 (− 0.34; 0.04)
LDL- C (mmol/L)	3.4 ± 0.8	3.7 ± 1.0	0.3 ± 0.8	3.3 ± 0.9	3.2 ± 0.6	− 0.1 ± 0.7	3.6 ± 0.9	3.5 ± 0.8	− 0.1 ± 0.4	− 0.03 (− 0.07; 0.00)	− 0.03 (− 0.07; − 0.00)*	− 0.00 (− 0.00; 0.07)
HDL- C (mmol/L)	1.2 ± 0.3	1.2 ± 0.3	0.0 ± 0.2	1.2 ± 0.2	1.2 ± 0.2	0.0 ± 0.2	1.3 ± 0.3	1.3 ± 0.3	0.0 ± 0.2	− 0.00 (− 0.01; 0.01)	0.00 (− 0.01; 0.01)	− 0.00 (− 0.02; 0.01)
Triglycerides (mmol/L)^+^	1.8 ± 0.9	1.9 ± 0.9	0.1 ± 1.2	1.4 ± 0.7	1.5 ± 0.6	0.1 ± 0.5	1.9 ± 1.0	1.7 ± 0.7	− 0.2 ± 0.6	− 0.00 (− 0.05; 0.04)	− 0.02 (− 0.07; 0.03)	0.02 (− 0.01; 0.05)
sCD163 (ng/L)	1.01 ± 0.4	0.95 ± 0.4	− 0.06 ± 0.3	0.95 ± 0.3	1.20 ± 0.4	0.25 ± 0.5	0.94 ± 0.5	1.01 ± 0.2	0.06 ± 0.4	− 0.01 (− 0.03;0.01)	− 0.00 (− 0.02;0.02)	− 0.01 (− 0.03;0.02)
TNF- α (pg/ml)^+^	0.34 ± 0.59	0.76 ± 0.60†	− 0.42 ± 0.7	0.29 ± 0.49	0.52 ± 0.62	0.23 ± 0.8	0.46 ± 0.60	0.65 ± 0.61	0.19 ± 0.8	− 0.02 (− 0.06; 0.03)	− 0.02 (− 0.06; 0.02)	0.00 (− 0.05; 0.05)
IL-6 (pg/ml)	18.7 ± 13.1	25.7 ± 14.6†	7.0 ± 17.3	25.4 ± 15.9	21.8 ± 11.8	− 3.6 ± 16.4	17.8 ± 15.1	16.2 ± 8.9	− 1.6 ± 12.7	− 0.89 (− 1.77; − 0.01)*	− 0.72 (− 1.53; 0.09)	− 0.17 (− 1.02; 0.68)
CRP (nmol/L)^+^	47.2 ± 36.3	41.8 ± 34.7	− 5.4 ± 35.2	33.3 ± 27.7	46.4 ± 35.0	13.1 ± 29.9	41.0 ± 31.7	42.6 ± 36.6	1.6 ± 58.3	1.54 (− 0.12; 3.20)	0.58 (− 2.23; 3.39)	0.95 (− 1.82; 3.74)
Cortisol (nmol/L)^+^	457.3 ± 210.8	454.3 ± 234.6	− 2.9 ± 334.6	548.7 ± 309.3	485.9 ± 415.8	− 62.8 ± 474.3	559.9 ± 192.7	431.5 ± 129.2	− 128.5 ± 171	− 4.98 (− 26.9; 16.9)	− 10.5 (− 24.1; 3.1)	5.47 (− 14.7; 25.7)
WBFI (kg/m^2^)	10.6 ± 3.4	10.8 ± 3.5	0.2 ± 0.9	10.6 ± 3.5	10.3 ± 3.5	− 0.3 ± 0.9	9.9 ± 4.0	9.5 ± 3.9	− 0.3 ± 0.8	− 0.04 (− 0.09; 0.01) *	− 0.04 (− 0.08; 0.01)	− 0.01 (− 0.06; 0.04)
AFI (kg/m^2^)	1.0 ± 0.4	1.0 ± 0.3	0.0 ± 0.2	1.1 ± 0.4	1.0 ± 0.4	− 0.1 ± 0.2	1.0 ± 0.4	0.9 ± 0.4	− 0.1 ± 0.1	− 0.01 (− 0.02; 0.00)	− 0.00 (− 0.01; 0.00)	− 0.01 (− 0.01; 0.00)
WC (cm)	105.4 ± 11.2	104.6 ± 11.3	− 0.8 ± 3.6	103.5 ± 12.2	103.1 ± 12.2	− 0.4 ± 3.7	103.3 ± 15.7	102.9 ± 15.4	− 0.4 ± 3.6	0.04 (− 0.16; 0.23)	0.04 (− 0.16; 0.24)	0.00 (− 0.22; 0.22)
Weight (kg)	85.9 ± 15.7	85.6 ± 15.4	− 0.3 ± 3.6	82.0 ± 13.8	81.8 ± 13.9	− 0.2 ± 3.5	84.2 ± 19.2	83.9 ± 18.6	− 0.3 ± 3.1	0.01 (− 0.18; 0.19)	− 0.00 (− 0.18; 0.18)	0.01 (− 0.19; 0.20)

Betas coefficients are presented as unstandardized coefficients adjusted for age, sex and baseline MVPA with the respective 95% confidence intervals

*AFI* android fat index, *CRP* C-reactive protein, *HIIT* high-intensity interval training, *HDL-C* high-density lipoprotein cholesterol, *IL-6* interleukin 6; LDL-C low-density lipoprotein cholesterol, *MCT* moderate continuous training, *TC* total cholesterol, *TNF-α* tumor necrosis factor alfa, *WBFI* whole-body fat index

^*^ Between-group changes significant at p < 0.05

† Within-group changes significant at p < 0.05

^+^Skewed values are presented as median ± inter quartile range

**Fig. 2:**
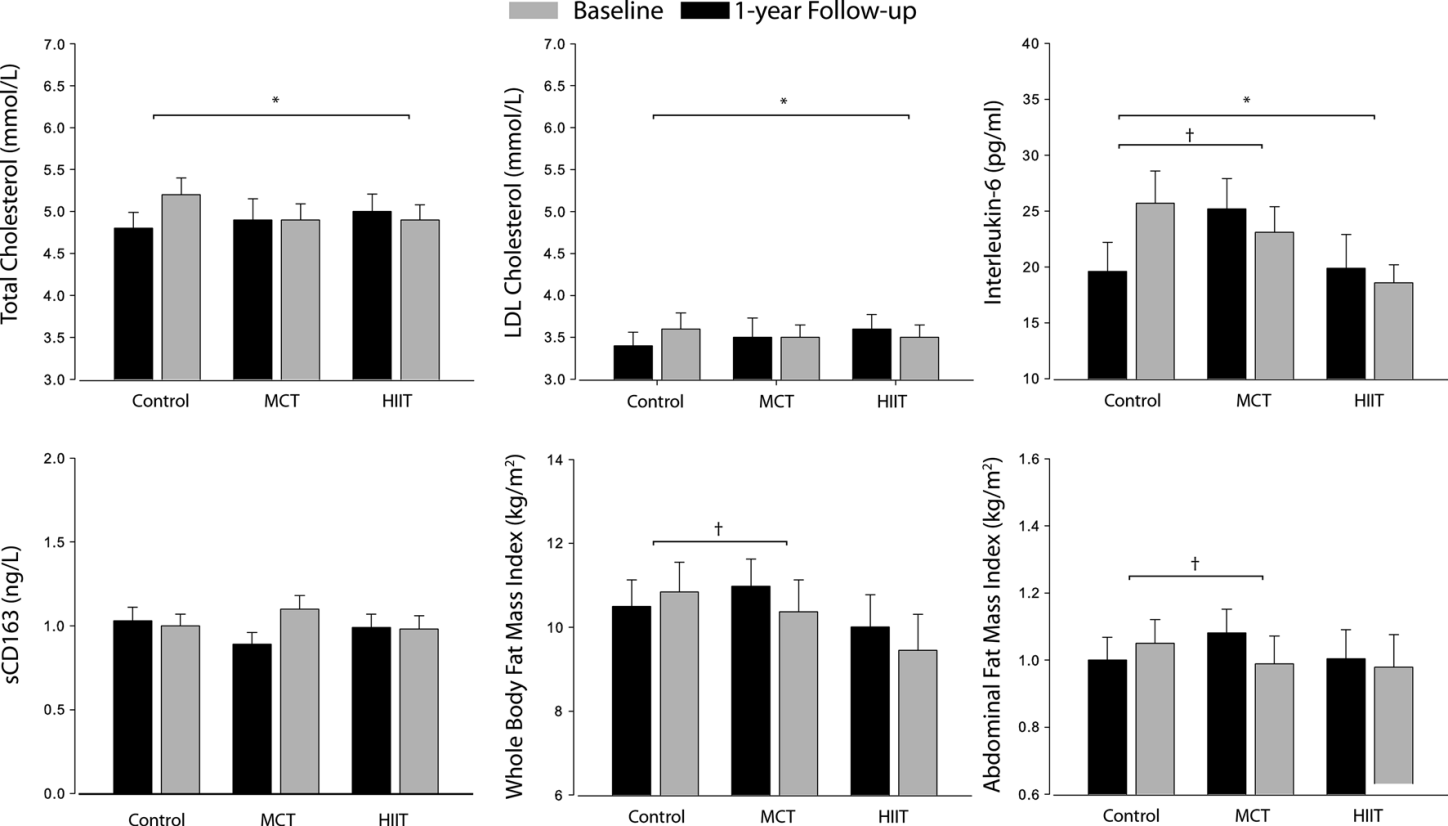
Intention-to-treat analysis derived from the GEE mixed model of the 1-year exercise intervention on total cholesterol, LDL colestherol, IL-6, sCD163, whole body fat and abdominal fat index. Absolute mean values and standard errors of means are presented, with models adjusted for sex and baseline MVPA. Abbreviations: HIIT, high-intensity interval training; LDL-C, low-density lipoprotein cholesterol; MCT, moderate continuous training. ^*^ HIIT vs. control group changes significant at p < 0.05; † MCT vs. control group changes significant at p < 0.05

Figure 2 depicts data from the absolute values on baseline and 1-year follow-up for the C-LDL, total cholesterol, IL-6, sCD163, WBFI and AFI using the ITTA.

## Discussion

To the best of our knowledge, this is the first RCT analysing the long-term impact of combined training with different intensities, on lipid and inflammatory profile in individuals with T2DM. The main finding was that long-term HIIT aerobic exercise, while combined with RT, can be used to prevent further derangement of total cholesterol, LDL-C and IL-6 in individuals with T2DM. Regarding the MCT group, we observed favourable changes only on IL-6, with no impact on the lipid profile after 1-year of intervention. Finally, there was no statistically significant difference in any of the biomarkers measured when comparing HIIT with RT group with the MCT with RT group.

Low-grade systemic inflammation has been independently implicated in metabolic disorders, such as insulin resistance and T2DM, and is typically presented with elevated levels of pro-inflammatory cytokines (i.e. IL-6, TNF-α, and CRP) [28, 29]. On the other hand, exercise can be used to prevent or counter the detrimental metabolic effects of elevated pro-inflammatory cytokines on different organs and tissues of the body [13]. In our investigation, both the MCT and HIIT combined with RT regimens showed favourable changes in the circulating levels of IL-6 following 1-year of exercise when compared with the control group, although no results were observed for the remaining inflammatory markers. The number of investigations that have analysed the impact of HIIT on the inflammatory profile in T2DM are scarce, with only two short-term interventions (< 12-week duration) reporting no substantial effects on IL-6, TNF-α, and CRP [19, 20].

Similar results to the ones in our investigation were found with reduced levels of IL-6, as well as CRP, while using a different protocol of high-intensity continuous training, either alone or combined with RT, in individuals with T2DM [30]. However, only the combination of aerobic and RT had a significant impact on the circulating levels of TNF-α.

Most of the benefits of exercise on the inflammatory profile seem to be mediated by body composition changes. For instance, without weight loss, a twice-weekly progressive aerobic program in patients with T2DM did not observe any changes in TNF-α and CRP levels following a 6-month intervention [31], which follows our findings. On the other hand, a 1-year aerobic exercise intervention plus weight loss [32] showed a significant reduction in both TNFα and CRP. Moreover, in overweight/obese individuals without T2DM, short and medium-term interventions have also observed a reduction in IL-6 following 2 weeks [33], and 16 weeks of HIIT [34], where modest weight loss was observed.

Beyond weight loss and the reduction in visceral fat mass, there are other proposed mechanisms for the improvements in the inflammatory profile following an exercise intervention [13, 29]. In fact, it is possible that the larger effect size for improvements in IL-6 observed in the MCT group combined with RT compared to the HIIT group combined with RT could be due to decreases in AFI and WBFI, which was not observed in the HIIT group. Nevertheless, the HIIT group still had a time-by-group interaction in IL-6 compared to the controls, regardless of body fat loss. Thus, it is possible that other mechanisms are responsible for the favourable changes in IL-6, such as the increased production of muscular anti-inflammatory myokines and the reduction of human monocyte Toll-like receptors 2 with exercise [35–37].

Another important finding from this investigation concerns the long-term impact of different exercise permutations on the sCD163 biomarker. Our results suggest that regardless of the exercise group there were no changes on the sCD163. The sCD163 molecule is increased after macrophage activation, with individuals with higher levels of adipose tissue having higher expression of this biomarker [38]. In fact, sCD163 has been positively associated with, obesity [6] and T2DM [7]. However, little is known about the effects of exercise on sCD163, with no study addressing the impact of different exercise intensities on this marker in individuals with T2DM. In individuals with non-alcoholic liver disease, a 3-month lifestyle intervention program, with both PA and dietary counselling, reduced the levels of sCD163 [39]. In contrast, decreased values have only been observed with dietary-induced weight loss [6], suggesting that sCD163 changes are dependent on body weight loss, particularly that of adipose tissue, which leads to a reduction of infiltrated active macrophages. Nevertheless, even with total and abdominal body fat reduction, as previously reported in the main findings of this study [22], the MCT group did not display corresponding decreases in sCD163 following the 1-year intervention. Future studies are warranted to further understand relationships among exercise training, sCD163, and adiposity in individuals with T2DM.

Regular exercise has also been shown to improve the lipid profile in individuals with T2DM [40, 41]. In our investigation, only the HIIT group observed significant changes in the circulating levels of LDL-C and total cholesterol compared to controls, whereas no changes were observed for the MCT group after 1-year of intervention. A recent meta-analysis in individuals with T2DM, examined the effects of HIIT and MCT on several biomarkers, including the lipid profile [42]. The analysis suggested that there were no differences between higher and moderate exercise intensities. However, the results were inconsistent between studies, with two reporting no effects of HIIT and MCT on LDL-C, HDL-C, total cholesterol and triglycerides [43, 44], and only one study observing changes in HDL-C and LDL-C with both HIIT and MCT, and decreased total cholesterol with [16]. The differences between these results and those reported in the present investigation may be explained by the baseline values of the participants, with most of our participants having relatively normal ranges of total cholesterol, HDL-C, and LDL-C levels, which did not change greatly after exercise. However, the biggest impact of the HIIT group on the lipid profile lies in differences against the control group, who incurred in adverse changes in their lipid profile over the course of 1-year without any exercise intervention. Moreover, the exercise protocols differed substantially between studies [16, 43, 44], with none of the interventions using the 1:1 (active-to-rest period ratio) protocol on a cycle ergometer and none of the interventions having a duration longer than 16-weeks. Another possible confounding factor is the baseline MVPA values (which were higher in the HIIT group), since it has been shown that higher physical activity intensities may be related with a more favourable lipid profile [45]. However, both HIIT and MCT had similar frequencies of patients fulfilling the PA guidelines and similar levels of cardiorespiratory fitness at baseline. Moreover, the results of the intervention remained the same after adjusting the models for baseline MVPA.

Given the results of our previous findings on vascular function [46], where the HIIT combined with RT had superior improvements when compared to MCT, we can speculate that the favourable changes in the lipid and inflammatory profile observed in the HIIT combined with RT group may have been mediated by some of these changes, alongside with other mechanism already addressed in the previous manuscript [46]. Nonetheless, even though we have shown that HIIT can be a feasible option for long-term clinical interventions (1-year), it maybe not be a viable solution in a more ecological setting given the physiological and psychological burden associated with long-term HIIT [21]. Thus, on the long run HIIT may be better suited in combination with intermittent periods of lower intensity trainings such as MCT to increase participant’s exercise adherence and enjoyment.

Despite the encouraging results observed, there are limitations in the present investigation that should be addressed. First, given that the sample size calculation for the D2FIT study was based off of the primary study outcome (i.e. HbA1c difference) and that for many inflammatory markers, such as that of CRP, as well as many of the lipid indices are known to exhibit high within-individual variability [47], it is likely that the lack of exercise intervention effect observed on many of the lipid and inflammatory parameters was due to small sample size. Another limitation involves t he lack of mid-term assessments during the 1-year intervention period, which did not allow for the assessment of the evolution of the inflammatory and lipid profile, especially in the first months of the intervention, where the adherence to the exercise programs may be higher. In addition, energy intake was not controlled during the intervention, which might have affected the lipid profile. As far as the strengths of our investigation are concerned, we used a 1-year intervention with supervised exercise sessions, which provides, for the first time, information on the long-term implications of combined HIIT and MCT protocols combined with RT in patients with T2DM.

## Conclusions

In conclusion, 1-year of combined training with MCT and HIIT induced favourable changes in circulating levels of IL-6, which may reflect the prevention of further derangement in the low-grade systemic inflammation typical in individuals with T2DM. Nonetheless, only HIIT was effective in attenuating the increases observed in the control group for both total cholesterol and LDL-C. Therefore, HIIT may be considered an effective strategy and an alternative to the traditional MCT guidelines, with similar or even greater long-term benefits, for improving inflammatory and lipid profiles in individuals with T2DM.

## Data Availability

The datasets used during the current study are available from the corresponding author on reasonable request.

## Abbreviations

AFI: Abdominal fat index
CVD: Cardiovascular disease
CRP: C-reactive protein
Hba1c: Glycated haemoglobin
scd163: Haptoglobin-hemoglobin receptor CD163
HRR: Heart rate reserve
HIIT: High-intensity interval training
ITTA: Intention-to-treat analysis
IL-6: Interleukin-6
MCT: Moderate continuous training
MVPA: Moderate-to-vigorous PA
PPA: Per-protocol analysis
PA: Physical activity
RCT: Randomized control trials
RT: Resistance training
TNF-α: Tumour necrosis factor-α
T2DM: Type 2 diabetes
WBFI: Whole-body fat index
